# Decomposition of sources of income-related health inequality applied on SF-36 summary scores: a Danish health survey

**DOI:** 10.1186/1477-7525-4-53

**Published:** 2006-08-22

**Authors:** Jens Gundgaard, Jørgen Lauridsen

**Affiliations:** 1 Institute of Public Health – Health Economics, University of Southern Denmark, JB Winsløws Vej 9, 5000 Odense C, Denmark; 2 Department of Economics and Business, University of Southern Denmark, Campusvej 55, 5230 Odense M, Denmark

## Abstract

**Background:**

If the SF-36 summary scores are used as health status measures for the purpose of measuring health inequality it is relevant to be informed about the sources of the inequality in order to be able to target the specific aspects of health with the largest impact.

**Methods:**

Data were from a Danish health survey on health status, health behaviour and socio-economic background. Decompositions of concentration indices were carried out to examine the sources of income-related inequality in physical and mental health, using the physical and mental health summary scores from SF-36.

**Results:**

The analyses show how the different subscales from SF-36 and various explanatory variables contribute to overall inequality in physical and mental health. The decompositions contribute with information about the importance of the different aspects of health and off-setting effects that would otherwise be missed in the aggregate summary scores. However, the complicated scoring mechanism of the summary scores with negative coefficients makes it difficult to interpret the contributions and to draw policy implications.

**Conclusion:**

Decomposition techniques provide insights to how subscales contribute to income-related inequality when SF-36 summary scores are used.

## Background

Equality in health is among the main objectives of health policy in many countries [1-3]. The present study considers the SF-36 instrument which is frequently used in health assessments or in health surveys to monitor health outcome as health-related quality of life (HRQoL). SF-36 has become one of the most widely used measures of health status [4,5], and has also been used in studies of health inequalities [6-10]. The SF-36 consists of 8 scales for different dimensions of health. The 8 scales can be summarised into two summary scores for physical and mental health, respectively. If the summary scores are used as health status measures for the purpose of measuring inequality indices, it is relevant to be informed about the sources of health and inequality in health in order to be able to target the specific aspects of health with the largest potential impacts. The objective of this paper is to apply decomposition techniques to the two summary scores from SF-36 when concentration indices are used as measures for income-related inequality in health.

The analyses of the study follow the lines of Clarke et al. [11], Wagstaff et al. [12] and Lauridsen et al. [13]. Clarke et al. [11] decompose a concentration index by dimension and subgroup separately. In Wagstaff et al. [12] a multivariate regression approach is used for a decomposition of background characteristics. The regression approach assists a decomposition of a single characteristic's impact on inequality in a health component into 1) its regressive impact on the variation in the health component, and 2) the impact due to income-related inequality in the characteristic itself. In Lauridsen et al. [13] the decomposition by dimension from Clarke et al. [11] is merged with the regression approach from Wagstaff et al. [12]. The concentration indices are each decomposed into the different dimensions of health summing up to the respective index and the effect on health from different socio-economic characteristics. Lauridsen et al. [13] apply the decomposition on 15D summary scores from a Finnish survey. The analysis shows that the different components of health contribute to health and inequality in health to varying degree, and that relationships to socio-economic and socio-demographic characteristics vary considerably.

To summarise, the present study adds to the literature by showing how to apply the methodology of Lauridsen et al. [13] to Physical Component Score (PCS) and Mental Component Score (MCS) values of the SF-36. The method reveals how the different HRQoL dimensions and background characteristics contribute to overall inequality in physical and mental health-related quality of life.

## Methods

### Study participants

Five thousand people living in Funen County, Denmark aged 16–80 were drawn from The Centralised Civil Register to participate in a health survey on health status, health behaviour and socio-economic background. The sample was stratified with respect to municipalities and the data have been weighted by the reciprocals of the selection probabilities (taking unit-nonresponse into account). The data were gathered in the period from October 2000 through April 2001. An external response rate of 68 percent was obtained [14]. A number of the respondents had to be excluded due to item-nonresponse, leaving a final working sample of 2,767, or 55 percent. Gundgaard & Sørensen [14] performed a descriptive response/nonresponse analysis and found that the number of women and men are approximately equal in the working sample. The participants are on average slightly younger than the nonparticipants. Middle-aged are slightly more prone to participate than the younger or older groups [14].

Income was defined as previous year's gross income (gross of tax and deductibles) and measured as a categorical variable with 17 categories. The respondents were ranked according to their income category taking the sample weights into account. Within the categories the respondents were ranked randomly.

Health status was measured using the PCS and the MCS from SF-36, respectively [4,15-21]. The PCS and MCS were each calculated by standardising each of the eight dimensions from the Danish SF-36, multiplying each dimension by its respective factor score coefficient, summing and standardising to the American norm of a mean of 50 and a standard deviation of 10 as recommended in Ware et al. [22] and Bjorner et al. [15].

### Statistical analysis

Income-related inequality in health was measured by the concentration index. The concentration index is a generalised Gini coefficient and is a measure of how equal one variable (HRQoL) is distributed with respect to the ranking of another variable (income) [23-25]. The concentration index ranges between -1 and 1, and if it is positive then good health is concentrated among the higher income groups and vice versa. The concentration index can be estimated by ordinary least squares (OLS) regression and approximate standard errors and t-statistics are easily obtained [23].

Concentration indices were estimated for PCS and MCS respectively. To explain the sources of income-related inequality in health these two indices were decomposed into components from the different dimensions of SF-36 and from explanatory background variables. The decomposition into dimension were carried out as expressing the concentration indices for PCS and MCS as a weighted sum of concentration indices for the dimensions with the relative share of the HRQoL as weights [11]. The decomposition into explanatory variables was carried out by a multivariate regression approach as in Wagstaff et al. [12], where the concentration indices for PCS and MCS were expressed as weighted sums of the concentration indices for the explanatory variables with the health elasticities with respect to the explanatory variables as weights [12]. The two decomposition techniques were merged together as in Lauridsen et al. [13] The concentration indices were then each decomposed into the different dimensions of health summing up to the respective indices PCS and MCS and the effect on health from different socio-demographic, socio-economic, and life-style characteristics. The technical details of the decomposition can be found in the appendix.

## Results

Table 1 shows descriptive statistics and concentration indices with t-statistics for each of the eight individual scales and the overall score for PCS and MCS, respectively. The overall PCS is 51.80 with a standard deviation of 7.92 indicating that physical health status is slightly better than the American norm of 50. Furthermore the variation is also smaller as the American norm is a standard deviation of 10. The concentration index of physical health using PCS with respect to income is 0.013. However, the concentration indices of the different scales present a large variation. All indices are statistically significant. The largest contributors to the overall concentration for PCS index are Physical Functioning, Role-Physical, and Bodily Pain. The MCS of 56.08 is somewhat better than the American norm of 50. The differential is bigger than half the standard deviation of 10 which is often considered to be the minimally important difference in HRQoL studies [26]. The variation is also smaller than the American counterpart. The income-related inequality in mental health status is lower than that of physical health status, as the overall concentration index for MCS is 0.008. The largest contributors to the overall concentration index for MCS are Role-Emotional and Mental Health.

**Table 1:** Descriptive statistics and concentration indices of PCS and MCS and each of its dimensions

					**PCS**			**MCS**		
						
	**Mean**	**SD**	**C**_*i*_	t*	**Weight**	**Contr**	**Contr (%)**	**Weight**	**Contr**	**Contr (%)**
Physical Function (PF)	93.24	14.55	0.017	9.56	0.333	0.006	44.4	-0.167	-0.003	-36.6
Role-Physical (RP)	87.47	28.65	0.026	6.84	0.175	0.005	35.4	-0.057	-0.001	-18.9
Bodily Pain (BP)	83.18	24.42	0.019	5.37	0.216	0.004	31.4	-0.061	-0.001	-14.6
General Health Perception (GH)	75.63	15.39	0.015	6.16	0.181	0.003	21.1	-0.011	0.000	-2.0
Vitality Scale (VT)	74.40	20.23	0.019	6.10	0.020	0.000	2.9	0.150	0.003	36.0
Social Function (SF)	95.57	13.74	0.007	4.25	-0.006	0.000	-0.3	0.205	0.001	18.2
Role-Emotional (RE)	91.49	24.09	0.018	5.89	-0.103	-0.002	-14.0	0.214	0.004	48.2
Mental Health (MH)	86.88	15.29	0.013	6.55	-0.205	-0.003	-20.8	0.418	0.005	69.7
PCS	51.80	7.92	0.013	7.10	1.000	0.013	100.0			
MCS	56.08	8.12	0.008	4.73				1.000	0.008	100.0

N = 2767; Contr – Contribution; PCS – Physical component score; MCS – Mental component score; *Heteroskedasticity-robust standard errors obtained to calculate t-statistics.

Table 2 shows the contribution from each subscale to the concentration index. The predicted concentration indices for PCS and MCS constitute 86.3 and 74.9 percent, respectively, of the observed concentration indices. The different subscales contribute according to the sign of their coefficient. This means that for most subscales the contributions to overall health point in opposite directions for PCS and MCS.

**Table 2:** Decompositions of PCS and MCS concentration indices into contributions from dimensions

		**PF**	**RP**	**BP**	**GH**	**VT**	**SF**	**RE**	**MH**	**Sum**
**PCS**	Predicted C	0.00522	0.00382	0.00319	0.00221	0.00028	-0.00004	-0.00141	-0.00220	0.01108
	Observed C	0.00570	0.00454	0.00403	0.00271	0.00037	-0.00004	-0.00180	-0.00267	0.01284
	Error CG	0.00048	0.00073	0.00084	0.00050	0.00009	-0.00001	-0.00039	-0.00048	0.00176
										
**MCS**	Predicted C	-0.00262	-0.00124	-0.00090	-0.00013	0.00211	0.00121	0.00294	0.00447	0.00585
	Observed C	-0.00286	-0.00147	-0.00114	-0.00016	0.00281	0.00142	0.00376	0.00544	0.00781
	Error CG	-0.00024	-0.00024	-0.00024	-0.00003	0.00070	0.00021	0.00082	0.00097	0.00196

N = 2767; PCS – Physical component score; MCS – Mental component score; PF – Physical Function; RP – Role-Physical; BP – Bodily Pain; GH – General Health Perception; VT – Vitality Scale; SF – Social Function; RE – Role-Emotional; MH – Mental Health (N = 2767).

The contributions from the different explanatory variables are shown in Tables 3 and 4 for PCS and MCS, respectively. As the contributions are rather small in absolute numbers, the contributions are shown in percentages of the overall predicted concentration indices. The different regressors contribute to the overall concentration index with various magnitudes and signs. For PCS the largest contributors are income and being retired. Also, the male 31–45 and 46–60 states are large contributors, however with negative signs. Furthermore, the educational regressors seem to play a role in the contribution to the overall inequality. Of the lifestyle variables, only a lifestyle with no exercises has a considerable contribution to the concentration index. For MCS, the largest contributors are being retired, being a white-collar worker (diminishes the inequality), being a young female (aged 16–30), and income. Also for MCS, the variable for no exercises plays a role in explaining inequality in health.

**Table 3:** Contribution from each regressor and each dimension to C of PCS (in percent of predicted C)

	**PF**	**RP**	**BP**	**GH**	**VT**	**SF**	**RE**	**MH**	**PCS**
ln(income)	25.07	8.62	22.81	14.17	1.58	-0.02	-6.62	-7.44	58.17
Male (31–45)	-3.21	-4.38	-9.16	-5.68	-0.53	0.04	0.72	3.63	-18.58
Male (46–60)	-7.62	-5.66	-7.98	-8.29	-0.19	0.02	1.23	0.09	-28.43
Male (61–70)	-0.44	-0.60	-0.58	-0.24	-0.08	0.01	0.16	0.51	-1.26
Male (71–80)	1.17	0.14	-0.53	-0.16	-0.12	0.01	0.66	1.05	2.23
Female (16–30)	-0.18	0.44	1.90	1.22	0.57	-0.07	-2.86	-4.49	-3.46
Female (31–45)	-0.72	-1.41	-2.64	-1.45	-0.27	0.02	0.44	1.55	-4.48
Female (46–60)	-0.11	-0.12	-0.19	-0.10	-0.01	0.00	0.01	0.07	-0.44
Female (61–70)	0.81	-0.60	-0.75	-0.54	-0.16	-0.01	-0.47	-0.16	-1.88
Female (71–80)	3.90	3.52	1.42	1.24	0.20	0.00	-0.17	-1.16	8.95
Low Education	0.17	0.10	0.15	-0.03	0.00	0.00	0.03	-0.01	0.41
Medium Education	-0.34	-1.12	-2.75	2.07	0.09	-0.01	-0.52	-1.45	-4.04
Other Education	2.07	9.92	7.32	2.12	0.01	-0.03	-1.62	-1.95	17.84
Skilled worker	-1.46	-1.84	-0.92	-0.43	-0.08	0.00	0.24	0.99	-3.49
White-collar worker	-3.87	-1.02	7.05	-1.00	-0.17	0.04	1.55	6.43	9.02
Selfemployed	-0.31	-0.23	0.96	-0.66	0.01	-0.01	-0.23	0.99	0.52
Assisting spouse	-0.04	0.18	0.05	0.04	0.01	0.00	-0.03	-0.05	0.16
Housewife	1.33	2.57	0.31	1.44	0.15	-0.03	-0.17	-2.00	3.60
Apprentice	0.59	0.28	0.70	-0.23	0.01	0.00	0.64	0.31	2.30
Student	0.83	-2.32	-2.50	-3.21	0.14	-0.03	2.14	-2.34	-7.28
Retired	28.02	24.08	15.63	18.38	1.18	-0.22	-5.73	-9.38	71.94
Unemployed	1.29	1.82	0.02	1.09	0.06	-0.03	-0.23	-1.17	2.84
Other job	-0.67	-0.78	-0.19	-0.43	-0.02	0.01	0.27	0.64	-1.17
Cohabitant	0.13	0.10	0.36	0.21	0.02	0.00	0.01	-0.09	0.74
Separated	-0.04	-0.12	-0.14	-0.01	-0.02	0.00	0.01	0.30	-0.02
Divorced	-0.56	-0.35	-0.07	-0.21	-0.04	0.01	0.25	0.34	-0.62
Widowed	0.15	-0.12	-0.35	-0.44	-0.02	-0.01	-1.31	-1.06	-3.15
Alone	-2.22	1.69	-3.06	-0.33	-0.06	0.00	-0.40	-2.60	-6.99
Other	0.02	0.03	-0.04	-0.06	0.00	0.00	-0.03	-0.01	-0.09
Daily smoker	0.13	0.29	0.56	0.39	0.05	-0.01	-0.11	-0.22	1.09
High alcohol	0.01	0.00	-0.15	-0.12	0.01	0.00	-0.06	-0.14	-0.46
Vegetables, cooked	-0.07	-0.39	-0.25	-0.08	0.02	0.00	-0.03	-0.12	-0.93
Vegetables, raw	0.24	0.03	0.16	0.29	0.07	0.00	0.01	-0.31	0.50
Fruit	0.09	-0.01	-0.13	0.03	0.00	0.00	-0.01	0.02	-0.02
No exercises	2.56	1.40	1.30	0.85	0.15	-0.02	-0.51	-0.71	5.03
Smoker and alcohol	0.02	-0.02	-0.01	0.01	0.01	0.00	-0.03	-0.09	-0.12
Smoke,alco,no exer	0.38	0.34	0.49	0.11	-0.03	0.00	0.07	0.20	1.57
Predicted C	47.11	34.46	28.80	19.95	2.52	-0.33	-12.70	-19.83	100.00

N = 2767; PF – Physical Function; RP – Role-Physical; BP – Bodily Pain; GH – General Health Perception; VT – Vitality Scale; SF – Social Function; RE – Role-Emotional; MH – Mental Health.

**Table 4:** Contribution from each regressor and each dimension to C of MCS (in percent of predicted C)

	**PF**	**RP**	**BP**	**GH**	**VT**	**SF**	**RE**	**MH**	**MCS**
ln(income)	-23.81	-5.30	-12.24	-1.56	22.58	1.31	26.19	28.66	35.84
Male (31–45)	3.05	2.70	4.92	0.63	-7.61	-2.71	-2.87	-13.98	-15.88
Male (46–60)	7.24	3.48	4.28	0.91	-2.79	-1.14	-4.87	-0.33	6.79
Male (61–70)	0.42	0.37	0.31	0.03	-1.17	-0.51	-0.64	-1.96	-3.16
Male (71–80)	-1.11	-0.09	0.28	0.02	-1.75	-0.71	-2.62	-4.04	-10.02
Female (16–30)	0.17	-0.27	-1.02	-0.13	8.23	4.43	11.31	17.29	40.00
Female (31–45)	0.68	0.87	1.42	0.16	-3.83	-1.10	-1.76	-5.96	-9.52
Female (46–60)	0.10	0.07	0.10	0.01	-0.15	-0.05	-0.05	-0.27	-0.23
Female (61–70)	-0.77	0.37	0.40	0.06	-2.31	0.40	1.86	0.63	0.64
Female (71–80)	-3.71	-2.16	-0.76	-0.14	2.85	-0.16	0.69	4.47	1.08
Low Education	-0.16	-0.06	-0.08	0.00	0.04	0.07	-0.11	0.03	-0.27
Medium Education	0.33	0.69	1.47	-0.23	1.27	0.51	2.07	5.59	11.71
Other Education	-1.96	-6.10	-3.93	-0.23	0.15	2.01	6.41	7.51	3.86
Skilled worker	1.38	1.13	0.49	0.05	-1.17	-0.22	-0.96	-3.81	-3.11
White-collar worker	3.67	0.63	-3.78	0.11	-2.45	-2.49	-6.15	-24.79	-35.26
Selfemployed	0.29	0.14	-0.51	0.07	0.21	0.91	0.93	-3.82	-1.79
Assisting spouse	0.04	-0.11	-0.03	0.00	0.15	-0.03	0.14	0.18	0.33
Housewife	-1.27	-1.58	-0.16	-0.16	2.20	1.85	0.67	7.72	9.27
Apprentice	-0.56	-0.17	-0.38	0.03	0.17	-0.25	-2.51	-1.21	-4.88
Student	-0.79	1.43	1.34	0.35	2.03	1.74	-8.48	9.02	6.64
Retired	-26.60	-14.80	-8.38	-2.03	16.85	13.79	22.69	36.16	37.68
Unemployed	-1.22	-1.12	-0.01	-0.12	0.81	1.88	0.93	4.50	5.65
Other job	0.64	0.48	0.10	0.05	-0.31	-0.84	-1.07	-2.46	-3.41
Cohabitant	-0.12	-0.06	-0.19	-0.02	0.30	-0.06	-0.05	0.35	0.15
Separated	0.04	0.07	0.08	0.00	-0.33	-0.11	-0.04	-1.16	-1.46
Divorced	0.53	0.21	0.04	0.02	-0.58	-0.70	-0.99	-1.30	-2.77
Widowed	-0.14	0.08	0.19	0.05	-0.22	0.83	5.17	4.08	10.03
Alone	2.11	-1.04	1.64	0.04	-0.92	0.13	1.60	10.00	13.56
Other	-0.01	-0.02	0.02	0.01	0.05	0.05	0.11	0.04	0.24
Daily smoker	-0.12	-0.18	-0.30	-0.04	0.76	0.45	0.45	0.86	1.88
High alcohol	-0.01	0.00	0.08	0.01	0.11	0.14	0.24	0.56	1.13
Vegetables, cooked	0.07	0.24	0.13	0.01	0.27	0.14	0.11	0.45	1.43
Vegetables, raw	-0.23	-0.02	-0.08	-0.03	0.97	0.18	-0.06	1.18	1.90
Fruit	-0.08	0.00	0.07	0.00	-0.02	0.10	0.05	-0.08	0.04
No exercises	-2.43	-0.86	-0.70	-0.09	2.09	1.08	2.01	2.72	3.81
Smoker and alcohol	-0.02	0.01	0.00	0.00	0.10	0.15	0.11	0.36	0.72
Smoke,alco,no exer	-0.36	-0.21	-0.26	-0.01	-0.45	-0.29	-0.29	-0.77	-2.64
Predicted C	-44.74	-21.18	-15.45	-2.20	36.15	20.76	50.24	76.42	100.00

N = 2767; PF – Physical Function; RP – Role-Physical; BP – Bodily Pain; GH – General Health Perception; VT – Vitality Scale; SF – Social Function; RE – Role-Emotional; MH – Mental Health.

## Discussion

The study reproduced the methods of Lauridsen et al. [13] in order to carry out decompositions of health status measures using the PCS and the MCS from SF-36, while Lauridsen et al. [13] applied 15D as health status measure.

The findings in Lauridsen et al. [13] were confirmed herein. That is, health status is a diversified matter, and an overall index may be too crude to health status for specific purposes. Policies combating inequalities in health might not produce any changes in the overall index if decreases in inequality in one type of health are offset by increases in another. Therefore, it is important to know the sources of health status and health inequality. For the specific dimensions of health the policies can be directed towards the distribution of the explanatory variables, modifying the relationship between the explanatory variables and health (with, for example, more health care or preventive measures targeted specific groups), or redistributing income between groups. It is important to note that the distribution of some of the explanatory variables are not modifiable (e.g. age, gender), and the estimated health effects of some characteristics are not necessarily applicable to all groups (e.g. due to self-selection). Furthermore, the basis for policy is also restricted by normative considerations.

Compared to 15D, the summary scores from SF-36 were not as straightforward to decompose. A summary score from SF-36 is complicated as the score is a function of eight other scores each building on several items. In the present analysis the eight SF-36 scores were taken as given, and there were no focus on the original items. In principle, the decomposition could have been carried out on the original items. However, decomposing a summary score into the different items might not have contributed with more relevant information. The relevant choice of level of decomposition depends on the focus of the analysis.

To correct for the confounding of physical and mental health, negative coefficients for some subscales subtract back the unwanted variance. This scoring mechanism has caused some controversy as a maximum score of PCS is achieved only when the mental health scales are at a low level and vice versa for MCS [19-21,27]. It is outside the scope of this article, however, to assess the scoring mechanism for the SF-36 summary scores. Nevertheless, the negative coefficients do make it harder to interpret the contributions to the decompositions as less inequality in some subscales tends to increase overall inequality. Furthermore, the negative coefficients result in contributions in opposite directions to the two summary scores. This means that policies combating inequalities in physical health, as measured by PCS, tend to worsen inequality in mental health, as measured by MCS, and vice versa.

## Conclusion

Decompositions of concentration indices with respect to the PCS and the MCS from SF-36 were carried out. When using SF-36 summary scores as health status measures the decompositions can be useful to reveal how the different subscales contribute to overall inequality. Furthermore, the decompositions allowed for explanatory variables to explain the sources of inequality. It was shown that the impact of socio-economic and health life style variables varied considerably. Income, gender, age, and being retired were the most important variables in explaining income-related inequality in physical and mental health. The decompositions also showed how the different subscales contributed to the PCS and the MCS. The decompositions into subscales turned out to be problematic as the complicated scoring mechanism of the summary scores produced contributions to inequality with opposite signs than expected.

## Competing interests

The study was carried out thanks to a research grant from The Health Insurance Foundation, Denmark (Sygekassernes Helsefond). The authors alone are responsible for the contents of the article. No financial or non-financial competing interests exist.

## Authors' contributions

Both authors participated in the design of the study, performed the statistical analyses, interpreted the results, and drafted the manuscript. Both authors read and approved the final manuscript.

## Appendix

Like most generic HRQoL measures [28] each of the PCS and MCS is comprised of dimensions that represent different aspects of health. Like several other indices the final health status measure is calculated as a sum of scores for each dimension, i.e. as Y=∑i=1IYi
 MathType@MTEF@5@5@+=feaafiart1ev1aaatCvAUfKttLearuWrP9MDH5MBPbIqV92AaeXatLxBI9gBaebbnrfifHhDYfgasaacH8akY=wiFfYdH8Gipec8Eeeu0xXdbba9frFj0=OqFfea0dXdd9vqai=hGuQ8kuc9pgc9s8qqaq=dirpe0xb9q8qiLsFr0=vr0=vr0dc8meaabaqaciaacaGaaeqabaqabeGadaaakeaacqWGzbqwcqGH9aqpdaaeWbqaaiabdMfaznaaBaaaleaacqWGPbqAaeqaaaqaaiabdMgaPjabg2da9iabigdaXaqaaiabdMeajbqdcqGHris5aaaa@3852@, where *Y*_*i *_is the contribution to overall health from dimension *i*. The PCS and MCS of the SF-36 fit into this frame, as each of them can be written as

Yj=50+10Yj(raw)=50+10∑i=18ajiYi(Z)=50+10∑i=18ajiYi−bici=(50−10∑i=18ajibici)+∑i=1810ajiciYi,j=PCS,MCS=νj0Y0+∑i=18νjiYi=∑i=08νjiYi     (1)
 MathType@MTEF@5@5@+=feaafiart1ev1aaatCvAUfKttLearuWrP9MDH5MBPbIqV92AaeXatLxBI9gBaebbnrfifHhDYfgasaacH8akY=wiFfYdH8Gipec8Eeeu0xXdbba9frFj0=OqFfea0dXdd9vqai=hGuQ8kuc9pgc9s8qqaq=dirpe0xb9q8qiLsFr0=vr0=vr0dc8meaabaqaciaacaGaaeqabaqabeGadaaakeaafaqaaeWacaaabaGaemywaK1aaSbaaSqaaiabdQgaQbqabaGccqGH9aqpcqaI1aqncqaIWaamcqGHRaWkcqaIXaqmcqaIWaamcqWGzbqwdaqhaaWcbaGaemOAaOgabaGaeiikaGIaemOCaiNaemyyaeMaem4DaCNaeiykaKcaaOGaeyypa0JaeGynauJaeGimaaJaey4kaSIaeGymaeJaeGimaaZaaabCaeaacqWGHbqydaWgaaWcbaGaemOAaOMaemyAaKgabeaaaeaacqWGPbqAcqGH9aqpcqaIXaqmaeaacqaI4aaoa0GaeyyeIuoakiabdMfaznaaDaaaleaacqWGPbqAaeaacqGGOaakcqWGAbGwcqGGPaqkaaaakeaaaeaacqGH9aqpcqaI1aqncqaIWaamcqGHRaWkcqaIXaqmcqaIWaamdaaeWbqaaiabdggaHnaaBaaaleaacqWGQbGAcqWGPbqAaeqaaaqaaiabdMgaPjabg2da9iabigdaXaqaaiabiIda4aqdcqGHris5aOWaaSaaaeaacqWGzbqwdaWgaaWcbaGaemyAaKgabeaakiabgkHiTiabdkgaInaaBaaaleaacqWGPbqAaeqaaaGcbaGaem4yam2aaSbaaSqaaiabdMgaPbqabaaaaOGaeyypa0JaeiikaGIaeGynauJaeGimaaJaeyOeI0IaeGymaeJaeGimaaZaaabCaeaadaWcaaqaaiabdggaHnaaBaaaleaacqWGQbGAcqWGPbqAaeqaaOGaemOyai2aaSbaaSqaaiabdMgaPbqabaaakeaacqWGJbWydaWgaaWcbaGaemyAaKgabeaaaaaabaGaemyAaKMaeyypa0JaeGymaedabaGaeGioaGdaniabggHiLdGccqGGPaqkcqGHRaWkdaaeWbqaamaalaaabaGaeGymaeJaeGimaaJaemyyae2aaSbaaSqaaiabdQgaQjabdMgaPbqabaaakeaacqWGJbWydaWgaaWcbaGaemyAaKgabeaaaaaabaGaemyAaKMaeyypa0JaeGymaedabaGaeGioaGdaniabggHiLdGccqWGzbqwdaWgaaWcbaGaemyAaKgabeaakiabcYcaSaqaaiabdQgaQjabg2da9iabbcfaqjabboeadjabbofatjabcYcaSiabb2eanjabboeadjabbofatbqaaiabg2da9GGaciab=17aUnaaBaaaleaacqWGQbGAcqaIWaamaeqaaOGaemywaK1aaSbaaSqaaiabicdaWaqabaGccqGHRaWkdaaeWbqaaiab=17aUnaaBaaaleaacqWGQbGAcqWGPbqAaeqaaaqaaiabdMgaPjabg2da9iabigdaXaqaaiabiIda4aqdcqGHris5aOGaemywaK1aaSbaaSqaaiabdMgaPbqabaGccqGH9aqpdaaeWbqaaiab=17aUnaaBaaaleaacqWGQbGAcqWGPbqAaeqaaaqaaiabdMgaPjabg2da9iabicdaWaqaaiabiIda4aqdcqGHris5aOGaemywaK1aaSbaaSqaaiabdMgaPbqabaaakeaaaaGaaCzcaiaaxMaadaqadiqaaiabigdaXaGaayjkaiaawMcaaaaa@CD41@

where *Y*_0 _= 1 and *Y*_1_, .., *Y*_8 _are the raw scores on the 8 items. The income-related inequality for each of the items is measured by the concentration index *C*_*i*_. If *Y*_*i *_can be explained linearly by *K *regressors through linear regression then the concentration index can be decomposed into contributions from the regressors as

Ci=∑k=1KδikμkμiCk+2μin∑n=1Nεi(n)R(n)=∑k=1KηikCk+1μiCGεi,     (2)
 MathType@MTEF@5@5@+=feaafiart1ev1aaatCvAUfKttLearuWrP9MDH5MBPbIqV92AaeXatLxBI9gBaebbnrfifHhDYfgasaacH8akY=wiFfYdH8Gipec8Eeeu0xXdbba9frFj0=OqFfea0dXdd9vqai=hGuQ8kuc9pgc9s8qqaq=dirpe0xb9q8qiLsFr0=vr0=vr0dc8meaabaqaciaacaGaaeqabaqabeGadaaakeaacqWGdbWqdaWgaaWcbaGaemyAaKgabeaakiabg2da9maaqahabaWaaSaaaeaaiiGacqWF0oazdaWgaaWcbaGaemyAaKMaem4AaSgabeaakiab=X7aTnaaBaaaleaacqWGRbWAaeqaaaGcbaGae8hVd02aaSbaaSqaaiabdMgaPbqabaaaaaqaaiabdUgaRjabg2da9iabigdaXaqaaiabdUealbqdcqGHris5aOGaem4qam0aaSbaaSqaaiabdUgaRbqabaGccqGHRaWkdaWcaaqaaiabikdaYaqaaiab=X7aTnaaBaaaleaacqWGPbqAaeqaaOGaemOBa4gaamaaqahabaGae8xTdu2aaSbaaSqaaiabdMgaPjabcIcaOiabd6gaUjabcMcaPaqabaaabaGaemOBa4Maeyypa0JaeGymaedabaGaemOta4eaniabggHiLdGccqWGsbGudaWgaaWcbaGaeiikaGIaemOBa4MaeiykaKcabeaakiabg2da9maaqahabaGae83TdG2aaSbaaSqaaiabdMgaPjabdUgaRbqabaaabaGaem4AaSMaeyypa0JaeGymaedabaGaem4saSeaniabggHiLdGccqWGdbWqdaWgaaWcbaGaem4AaSgabeaakiabgUcaRmaalaaabaGaeGymaedabaGae8hVd02aaSbaaSqaaiabdMgaPbqabaaaaOGaem4qamKaem4raC0aaSbaaSqaaiab=v7aLnaaBaaameaacqWGPbqAaeqaaaWcbeaakiabcYcaSiaaxMaacaWLjaWaaeWaceaacqaIYaGmaiaawIcacaGLPaaaaaa@7B1D@

where *δ*_*ik*_, *μ*_*k *_and *C*_*k *_are the OLS-coefficient, mean and concentration index of the *k*'th regressor [12], and *CG*_ε_/*μ*_*i *_is a residual component of the inequality that cannot be explained. Using that the concentration index of *ν*_*ji*_*Y*_*i *_is equal to the concentration index of *Y*_*i *_and that the concentration index of *Y*_0 _is equal to zero, the concentration index of *Y*_*j *_can also be decomposed into a weighted average[11]:

Cj=∑i=08wjiCi=∑i=18wjiCi,     (3)
 MathType@MTEF@5@5@+=feaafiart1ev1aaatCvAUfKttLearuWrP9MDH5MBPbIqV92AaeXatLxBI9gBaebbnrfifHhDYfgasaacH8akY=wiFfYdH8Gipec8Eeeu0xXdbba9frFj0=OqFfea0dXdd9vqai=hGuQ8kuc9pgc9s8qqaq=dirpe0xb9q8qiLsFr0=vr0=vr0dc8meaabaqaciaacaGaaeqabaqabeGadaaakeaacqWGdbWqdaWgaaWcbaGaemOAaOgabeaakiabg2da9maaqahabaGaem4DaC3aaSbaaSqaaiabdQgaQjabdMgaPbqabaaabaGaemyAaKMaeyypa0JaeGimaadabaGaeGioaGdaniabggHiLdGccqWGdbWqdaWgaaWcbaGaemyAaKgabeaakiabg2da9maaqahabaGaem4DaC3aaSbaaSqaaiabdQgaQjabdMgaPbqabaaabaGaemyAaKMaeyypa0JaeGymaedabaGaeGioaGdaniabggHiLdGccqWGdbWqdaWgaaWcbaGaemyAaKgabeaakiabcYcaSiaaxMaacaWLjaWaaeWaceaacqaIZaWmaiaawIcacaGLPaaaaaa@5111@

where *C*_*j *_is the concentration index for *Y*_*j*_, *C*_*i *_the concentration index for *Y*_*i*_, and *w*_*ij *_a weight attached to the *i*'th dimension, estimated as wji=νjiμiμj
 MathType@MTEF@5@5@+=feaafiart1ev1aaatCvAUfKttLearuWrP9MDH5MBPbIqV92AaeXatLxBI9gBaebbnrfifHhDYfgasaacH8akY=wiFfYdH8Gipec8Eeeu0xXdbba9frFj0=OqFfea0dXdd9vqai=hGuQ8kuc9pgc9s8qqaq=dirpe0xb9q8qiLsFr0=vr0=vr0dc8meaabaqaciaacaGaaeqabaqabeGadaaakeaacqWG3bWDdaWgaaWcbaGaemOAaOMaemyAaKgabeaakiabg2da9maalaaabaacciGae8xVd42aaSbaaSqaaiabdQgaQjabdMgaPbqabaGccqWF8oqBdaWgaaWcbaGaemyAaKgabeaaaOqaaiab=X7aTnaaBaaaleaacqWGQbGAaeqaaaaaaaa@3D50@, with *μ*_*j *_and *μ*_*i *_being the means of *Y*_*j *_and *Y*_*i *_respectively. Combining (2) and (3), the decomposition of *C*_*j *_follows as [13]

Ci=∑i=18wjiCi=∑i=18νjiμiμj[∑k=1KδikμkμiCk+1μiCGεi]=∑i=18∑k=1KνjiδikμkμjCk+∑j=1JνjiμjCGεi,j=PCS,MCS.     (4)
 MathType@MTEF@5@5@+=feaafiart1ev1aaatCvAUfKttLearuWrP9MDH5MBPbIqV92AaeXatLxBI9gBaebbnrfifHhDYfgasaacH8akY=wiFfYdH8Gipec8Eeeu0xXdbba9frFj0=OqFfea0dXdd9vqai=hGuQ8kuc9pgc9s8qqaq=dirpe0xb9q8qiLsFr0=vr0=vr0dc8meaabaqaciaacaGaaeqabaqabeGadaaakeaafaqaaeGabaaabaGaem4qam0aaSbaaSqaaiabdMgaPbqabaGccqGH9aqpdaaeWbqaaiabdEha3naaBaaaleaacqWGQbGAcqWGPbqAaeqaaaqaaiabdMgaPjabg2da9iabigdaXaqaaiabiIda4aqdcqGHris5aOGaem4qam0aaSbaaSqaaiabdMgaPbqabaGccqGH9aqpdaaeWbqaaGGaciab=17aUnaaBaaaleaacqWGQbGAcqWGPbqAaeqaaaqaaiabdMgaPjabg2da9iabigdaXaqaaiabiIda4aqdcqGHris5aOWaaSaaaeaacqWF8oqBdaWgaaWcbaGaemyAaKgabeaaaOqaaiab=X7aTnaaBaaaleaacqWGQbGAaeqaaaaakmaadmGabaWaaabCaeaadaWcaaqaaiab=r7aKnaaBaaaleaacqWGPbqAcqWGRbWAaeqaaOGae8hVd02aaSbaaSqaaiabdUgaRbqabaaakeaacqWF8oqBdaWgaaWcbaGaemyAaKgabeaaaaaabaGaem4AaSMaeyypa0JaeGymaedabaGaem4saSeaniabggHiLdGccqWGdbWqdaWgaaWcbaGaem4AaSgabeaakiabgUcaRmaalaaabaGaeGymaedabaGae8hVd02aaSbaaSqaaiabdMgaPbqabaaaaOGaem4qamKaem4raC0aaSbaaSqaaiab=v7aLnaaBaaameaacqWGPbqAaeqaaaWcbeaaaOGaay5waiaaw2faaaqaaiabg2da9maaqahabaWaaabCaeaadaWcaaqaaiab=17aUnaaBaaaleaacqWGQbGAcqWGPbqAaeqaaOGae8hTdq2aaSbaaSqaaiabdMgaPjabdUgaRbqabaGccqWF8oqBdaWgaaWcbaGaem4AaSgabeaaaOqaaiab=X7aTnaaBaaaleaacqWGQbGAaeqaaaaaaeaacqWGRbWAcqGH9aqpcqaIXaqmaeaacqWGlbWsa0GaeyyeIuoaaSqaaiabdMgaPjabg2da9iabigdaXaqaaiabiIda4aqdcqGHris5aOGaem4qam0aaSbaaSqaaiabdUgaRbqabaGccqGHRaWkdaaeWbqaamaalaaabaGae8xVd42aaSbaaSqaaiabdQgaQjabdMgaPbqabaaakeaacqWF8oqBdaWgaaWcbaGaemOAaOgabeaaaaaabaGaemOAaOMaeyypa0JaeGymaedabaGaemOsaOeaniabggHiLdGccqWGdbWqcqWGhbWrdaWgaaWcbaGae8xTdu2aaSbaaWqaaiabdMgaPbqabaaaleqaaaaakiabcYcaSiabdQgaQjabg2da9iabbcfaqjabboeadjabbofatjabcYcaSiabb2eanjabboeadjabbofatjabc6caUiaaxMaacaWLjaWaaeWaceaacqaI0aanaiaawIcacaGLPaaaaaa@B720@

As demonstrated by [13], the contribution from the *k*'th regressor to CjPRED
 MathType@MTEF@5@5@+=feaafiart1ev1aaatCvAUfKttLearuWrP9MDH5MBPbIqV92AaeXatLxBI9gBaebbnrfifHhDYfgasaacH8akY=wiFfYdH8Gipec8Eeeu0xXdbba9frFj0=OqFfea0dXdd9vqai=hGuQ8kuc9pgc9s8qqaq=dirpe0xb9q8qiLsFr0=vr0=vr0dc8meaabaqaciaacaGaaeqabaqabeGadaaakeaacqWGdbWqdaqhaaWcbaGaemOAaOgabaGaemiuaaLaemOuaiLaemyrauKaemiraqeaaaaa@33BF@ is then obtained as ∑i=18νjiδikμkμjCk
 MathType@MTEF@5@5@+=feaafiart1ev1aaatCvAUfKttLearuWrP9MDH5MBPbIqV92AaeXatLxBI9gBaebbnrfifHhDYfgasaacH8akY=wiFfYdH8Gipec8Eeeu0xXdbba9frFj0=OqFfea0dXdd9vqai=hGuQ8kuc9pgc9s8qqaq=dirpe0xb9q8qiLsFr0=vr0=vr0dc8meaabaqaciaacaGaaeqabaqabeGadaaakeaadaaeWbqaamaalaaabaacciGae8xVd42aaSbaaSqaaiabdQgaQjabdMgaPbqabaGccqWF0oazdaWgaaWcbaGaemyAaKMaem4AaSgabeaakiab=X7aTnaaBaaaleaacqWGRbWAaeqaaaGcbaGae8hVd02aaSbaaSqaaiabdQgaQbqabaaaaaqaaiabdMgaPjabg2da9iabigdaXaqaaiabiIda4aqdcqGHris5aOGaem4qam0aaSbaaSqaaiabdUgaRbqabaaaaa@45A3@, while the contribution from the *i*'th dimension is obtained as ∑k=1KνjiδikμkμjCk
 MathType@MTEF@5@5@+=feaafiart1ev1aaatCvAUfKttLearuWrP9MDH5MBPbIqV92AaeXatLxBI9gBaebbnrfifHhDYfgasaacH8akY=wiFfYdH8Gipec8Eeeu0xXdbba9frFj0=OqFfea0dXdd9vqai=hGuQ8kuc9pgc9s8qqaq=dirpe0xb9q8qiLsFr0=vr0=vr0dc8meaabaqaciaacaGaaeqabaqabeGadaaakeaadaaeWbqaamaalaaabaacciGae8xVd42aaSbaaSqaaiabdQgaQjabdMgaPbqabaGccqWF0oazdaWgaaWcbaGaemyAaKMaem4AaSgabeaakiab=X7aTnaaBaaaleaacqWGRbWAaeqaaaGcbaGae8hVd02aaSbaaSqaaiabdQgaQbqabaaaaaqaaiabdUgaRjabg2da9iabigdaXaqaaiabdUealbqdcqGHris5aOGaem4qam0aaSbaaSqaaiabdUgaRbqabaaaaa@45C8@.
